# A scoping review of best practices in home enteral tube feeding

**DOI:** 10.1017/S1463423622000366

**Published:** 2022-08-04

**Authors:** Cliona Byrnes, David Mockler, Linda Lyons, Dorothy Loane, Edel Russell, Annemarie E. Bennett

**Affiliations:** 1 Human Nutrition and Dietetics Student, Trinity College Dublin, Dublin, Ireland; 2 Trinity Centre for Health Sciences, St James’ Healthcare Campus, Dublin, Ireland; 3 Senior Community Dietitian, Health Service Executive, Navan, County Meath, Ireland; 4 Dietitian Manager, Health Service Executive, Dundalk, County Louth, Ireland

**Keywords:** community care, enteral nutrition, home enteral nutrition, home enteral tube feeding, nutrition support, primary care

## Abstract

**Aims:**

To review the experiences of healthcare professionals (HCPs) and service users on the provision and receipt of home enteral nutrition (HEN) in primary care settings, respectively.

**Backgrounds:**

HEN supports the nutritional needs of service users in primary care settings who are unable to meet their nutritional requirements through oral intake alone. While HEN supports service users to remain in their home, the provision of HEN services can be variable. The prevalence of HEN is increasing as health systems shift delivery of care from acute to primary care settings, and therefore the evolving needs of HCPs and service users in relation to HEN deserve exploration.

**Methods:**

Quantitative and qualitative studies were included if they described (1) practices that support best outcomes in adults on HEN and residing in their own homes and/or (2) service user and HCP experiences of HEN. Studies on the economics of HEN were included. Databases searched included MEDLINE/PubMed, EMBASE, Web of Science, and CINAHL. Publications up to March 2021 were included. A descriptive analytical approach was used to summarise the findings.

**Findings:**

Key themes included the importance of initial education to enable service users to adapt to HEN and the need for support from knowledgeable HCPs. Access to support from HCPs in primary care was limited, and some HCPs felt their knowledge of HEN was inadequate. Service users highlighted the significant impact of HEN on daily living and emphasised the need for support from a HEN team. HEN services were also associated with reduced hospital admissions, lengths of stay in hospital, and costs of hospitalisation.

**Conclusions:**

A specialist HEN service can manage enteral nutrition-related complications, reduce unnecessary hospital admissions, and improve quality of care and patient satisfaction. Further education of HCPs is needed on the provision of HEN.

## Introduction

Enteral nutrition (EN) or enteral tube feeding refers to the feeding of patients directly into the gastrointestinal tract via a feeding tube. Home enteral nutrition (HEN) or home enteral tube feeding (HETF) supports the nutritional needs of patients in a primary care setting who are unable to meet their nutritional requirements through oral intake alone. The ESPEN guidelines advise that a percutaneous endoscopic gastrostomy (PEG) is the preferred access device and should be placed when long-term HEN is required (Bischoff *et al.*, 2020). The prevalence of HEN has increased globally due to increased emphasis on moving care away from the more costly acute environment to the primary care setting (Ojo, 2015). EN is often initiated in an acute setting and continued as a long-term intervention on discharge. It can correct significant nutritional deficiencies, mitigate loss of body weight, and attenuate deterioration of the quality of life (QoL), all of which are linked to poor oral nutritional intake (Bischoff *et al.*, 2020). Importantly, HEN allows patients to remain in their typical social and family environment and reduces the incidence of infectious complications, number of hospital admissions, and healthcare costs (Kurien *et al.*, 2012, Hall *et al.*, 2014, Klek *et al.*, 2014, Mapson and Brookes, 2020).

The management of HEN in the primary care setting can be challenging and requires co-ordination of the patient, their caregivers, and the multidisciplinary team (Maher *et al.*, 2017). Furthermore, the availability of a dedicated HEN programme consisting of healthcare providers, including nurses, dietitians, and other allied health professionals, varies between countries and healthcare centres (Gramlich *et al.*, 2018). In Ireland, a study by Boland *et al.* found that HEN patients and carers prefer a single, expert point of contact to manage their nutritional needs and provide structured follow-up. A single point of contact after discharge may improve patient experiences and reduce avoidable hospital admissions, particularly for gastrostomy replacement (Boland *et al.*, 2017).

The lack of a dedicated HEN service means that gastrostomy aftercare is often fragmented between providers. Tube blockages are the most frequent gastrostomy complication, with tube dislodgement, over-granulation around the stoma site, and broken Y adaptors also posing issues (McNamara *et al.*, 2000, Kurien *et al.,* 2012, Johnson *et al.*, 2019). Complications have historically been dealt with by a range of providers, from primary care to emergency care providers and oncologists to various surgeons. This fragmented care has been associated with high costs, low patient satisfaction, and occasional loss of enteral access (Hall *et al.*, 2014). Some of the most common issues encountered by the HEN team include patient compliance with the prescribed HEN programme, mechanical issues of the tube site, and gastrointestinal problems (Johnson *et al.*, 2019). A community dietitian with an extended role in HEN can manage common tube and stoma-related complications, reducing the need for hospital or GP visits. A community dietitian can also revise feeding regimens for patients on HEN, reducing the need for a review by a hospital dietitian that may lessen the burden on dietetic outpatient waiting lists. For more complex cases, a shared care approach could be explored (O’Riordan *et al.*, 2020).

In the UK, a large prospective study (Kurien *et al.*, 2012) of a HEN team found that 227 hospital admissions were avoided in a caseload of 313 patients, over a one-year period. While 59 patients were admitted to hospital, only seven (12%) were due solely to an issue with the gastrostomy. Prior to the establishment of the HEN team, gastrostomy-related issues accounted for 23% of hospital readmissions. The study also noted that in addition to the cost savings, there was a positive impact on psychological well-being of service users by enabling them to remain at home.

The potential positive impact of HEN on service user outcomes is clear, although research on the cost-effectiveness of a dedicated HEN service is ongoing and emerging (Bischoff *et al.*, 2020). To the authors’ knowledge, this is the first scoping review that aims to collate the literature to date on the impact of HEN on service user and healthcare outcomes when a dedicated HEN service and associated best practice guidelines for establishing such a service are in place.

## Methods

While there is no universally accepted definition of a scoping review (Levac *et al.*, 2010, Pham *et al.*, 2014), a principal characteristic of this method is that it aims to provide an overview or map of the evidence of a broad topic (Arksey and O’Malley, 2005). The framework for conducting a scoping review, developed by Arksey and O’Malley (Arksey and O’Malley, 2005) and later refined by Levac *et al.* (Levac *et al.*, 2010), provides a rigorous and transparent method for mapping areas of research. A scoping review is suitable for research topics such as this as it allows for greater flexibility than traditional systematic reviews or meta-analyses and can account for a diversity of literature and studies (Arksey and O’Malley, 2005, Levac *et al.*, 2010, Pham *et al.*, 2014).

### Inclusion and exclusion criteria

Quantitative and qualitative studies were included if they described (1) practices that support best outcomes in adults on HEN and residing in their own homes and/or (2) service user and HCP experiences of HEN. Studies on health economics relevant to adult HEN patients residing in their own homes were also included.

Studies on nasogastric feeding, total parenteral nutrition, patients under 18 years of age on HEN, patients residing in residential care sites or private nursing homes, and patients residing in community houses for disabilities or residential care sites for disabilities were excluded. As quality assessment is not a priority in scoping reviews, studies were not excluded based on their quality.

### Search strategy

The search strategy (supplementary material) was developed using existing literature and with assistance from a medical librarian. The search strategy was applied to MEDLINE/PubMed, EMBASE, Web of Science, and CINAHL, using search terms such as EN, enteral feeding, tube feeding, intragastric feeding, intestinal feeding, percutaneous endoscopic gastrostomy, home, patient, health professional, and community care. The search strategy applied to EMBASE is in Box 1. The search included publications up to March 2021, with no minimum year of publication.

Box 1. Search strategy applied to EMBASE

Enteral Nutrition/

((Enteral OR enteric) adj2 (feeding OR nutrition)).ti,ab.

(intragastric feeding OR intestinal feeding OR tube feeding).ti,ab.

gastrostomy/

gastrostom*.ti,ab.

(percutaneous endoscopic gastrostomy OR PEG).ti,ab.

or/1-6

exp Community Health Services/

((Enteral OR enteric) adj2 (feeding OR nutrition) adj4 (home* OR communit*)).ti,ab.

((gastrostom* OR intragastric feeding OR intestinal feeding OR tube feeding OR percutaneous endoscopic gastrostomy OR PEG) adj4 (home* OR communit*)).ti,ab.

or/8-10

7 AND 11

### Analysis

Titles and abstract screening was completed in *Covidence* software. Review articles, commentaries, conference abstracts, and case reports were excluded. Screening was completed by two independent reviewers, and conflicts were managed and resolved by discussion between the two reviewers. The full text of the remaining studies was retrieved and reviewed. A ‘descriptive analytical’ approach in line with the Arksey and O’Malley (Arksey and O’Malley, 2005) framework was used to collate the findings in a consistent manner. A template was applied to all studies to extract the following data: country; sample size; ethnicity; target population; study type; data collection; and method of analysis. Data were extracted from quantitative and qualitative studies to facilitate the analysis of reported outcomes. Data were extracted by the first author after discussion of the approach with the second author. The entries were subsequently checked by the second author, and any disagreements were settled by consensus. Once extracted, the data were coded under themes. Themes developed from the reported findings of quantitative and qualitative studies and iteratively reviewed to ensure that the recurring points were accurately represented. The implications of this scoping review’s findings were considered in the context of current and future policy and practice. As per the advanced scoping review methodology (Levac *et al.*, 2010), stakeholders (three community dietitians) were consulted throughout the process, and the findings were discussed in depth at the end of the review.

## Results

The search retrieved 3,522 papers, with 2,147 papers eligible for screening once duplicates were removed. Screening was completed using Covidence (www.covidence.org ). A total of 339 abstracts were identified for detailed review, and 26 publications were selected for data extraction. Nineteen studies were related to the experience of HEN from the perspective of service users and HCPs, and seven studies were related to the economics of HEN.

Table 1 shows the main characteristics of studies (*n*19) included in the analysis that relate to service user and HCP experience of HEN. The majority (*n*13) were published after 2003. Three studies focused on the experience of HCPs and 16 focused on the experiences of service users.

**Table 1.** Study characteristics

Author (Year)	Country	Sample Size	Ethnicity	Study Population	Study Type	Data Collection	Analysis
Ang *et al.* (2019)	Singapore	*n*9	Chinese *n* = 8	Adults on long-term HEN^*^	Qualitative	Semi-structured interview	Inductive content
		(*n*3 male)	Indian *n* = 1				
Green *et al.* (2019)	UK	*n*19	Not reported	Adults on long-term HEN^*^	Qualitative	Semi-structured interview	Thematic
		(*n*11 male)					
Thomas *et al.* (2019)	UK	*n*15	White British *n*13	Adults on HEN	Qualitative	Semi-structured interview	Interpretative phenomenological
		(*n*10 male)	Non-white British *n*2				
Asiedu *et al.* (2018)	USA	*n*10	White, non-Hispanic	Adults on long-term HEN^*^	Qualitative	Photos and interviews	Inductive content
		(*n*6 male)					
Halliday *et al.* (2017)	UK	*n*15	White British	Patients who recently had surgery for oesophagogastric cancer	Qualitative	Semi-structured interviews	Inductive thematic
		(*n*12 male)					
Bjuresäter *et al.* (2015)	Sweden	*n*11	Not reported	Adults currently or recently on HEN	Qualitative	One-on-one interviews	Grounded theory
		(*n*5 male)					
Martin *et al.* (2012)	Sweden	*n*104	Not reported	Adults on long-term HEN	Quantitative	Questionnaire	Descriptive statistics
		(*n*67 male)					
Brotherton *et al.* (2007)	UK	Patients *n*15	Not reported	Adults with a PEG for at least 4 weeks^*^;	Semi-quantitative	Semi-structured interviews and questionnaires	Descriptive statistics
		(*n*6 male)		District nurses and dietitians identified from patient records			
		Dietitians *n*23					
		Nurses *n*18					
Madigan *et al.* (2007)	Northern Ireland	*n*23	Not reported	GPs in health boards in Northern Ireland	Qualitative	Semi-structured one-to-one interviews	Thematic
		(*n*20 male)					
Paccagnella *et al.* (2007)	Italy	*n*20	Not reported	Adults on long-term HEN^*^	Quantitative	Psychological tests and questionnaires	Descriptive statistics
		(*n*12 male)					
Brotherton *et al.* (2007)	UK	*n*15	Not reported	Adults with a PEG for at least 4 weeks^*^	Qualitative	Face-to-face semi-structured interviews	Thematic
		(*n*6 male)					
Jordan *et al.* (2006)	UK	*n*20	Not reported	Adults with a long-term PEG	Semi-qualitative	Structured and semi-structured interviews	Thematic
		(*n*10 male)					
Thompson *et al.* (2006)	USA	*n*12	Caucasian	Adults on long-term HEN	Qualitative	Questionnaire and semi-structured interviews	Grounded theory
		(*n*7 male)					
Liley *et al.* (2003)	UK	*n*6	Not reported	Adults with ≥ 12 weeks of experience of HEN^*^	Qualitative	Semi-structured interviews	Grounded theory
Loeser *et al.* (2003)	Germany	*n*155	Not reported	Adults on HEN for more than 4 weeks	Quantitative	Questionnaire	Descriptive statistics
		(*n*88 male)					
McNamara *et al.* (2001)	Republic of Ireland	GPs *n*80	Not reported	GPs in Dublin region, hospital dietitians discharging patients on HEN, employees from nutrition product companies	Quantitative	Questionnaire	Descriptive statistics
		Dietitians *n*77					
		Nutrition company employees *n*10					
Roberge *et al.* (2000)	France	*n*39	Not reported	Patients on HEN after treatment for head and neck or oesophageal cancer	Quantitative	Questionnaire	Descriptive statistics
		(*n*38 male)					
Schneider *et al.* (2000)	France	*n*38	Not reported	Adults on long-term HEN	Quantitative	Questionnaire	Descriptive statistics
		(*n*24 male)					
L’Estrange (1997)	Northern Ireland	*n*39	Not reported	All HEN patients on community dietitians’ register	Quantitative	Structured interview	Descriptive statistics
		(*n*20 male)					

* Carers also included in study population but have not been analysed as part of this scoping review.

Key findings are shown in Table 2. Four key themes emerged (Table 3), namely (1) importance of initial education, (2) impact of HEN and its most common complications on the day-to-day life of patients, (3) need for ongoing, structured support – routine and urgent – from a specialised service, and (4) impact of HEN on QoL.

**Table 2.** Summary of key findings

Author (Year)	Country	Study Aim	Key Findings
**PART A – Qualitative Studies**			
Ang *et al.* (2019)	Singapore	Understand patients’ and carers’ experience upon initiation of long-term HEN	Patient perspectives: Lack of knowledge about PEG insertion increased anxiety Access to a nutrition nurse and technical support was viewed positively
Green *et al.* (2019)	UK	Understand patients’ and carers’ experience of long-term HEN	Patient perspectives: More training on managing the ET would be welcome before leaving hospital Strong preference for ET-related issues to be managed at home if they arise Most common ET issues were dislodgement, stoma infection, and over-granulating tissue HEN significantly impacts on the management of everyday activities Access to support from HCPs was variable
Thomas *et al.* (2019)	UK	Establish the impact of HEN on daily life of those with a diagnosis of head and neck cancer	Patient perspectives: Knowledge and skill development enabled more effective adaptation to HEN Facilitating patient autonomy with managing the ET was important HEN significantly impacted everyday activities of daily living, e.g., meals, sleep, travel, work HEN curtailed social activities due to feeding duration and anxiety over damage to ET
Asiedu *et al.* (2018)	USA	Understand patients’ and carers’ experience of long-term HEN	Patient perspectives: Need for more specificity in educational material on what to expect with HEN, e.g., complications Ongoing education on practical logistics of HEN would be welcome Most common ET issues were leaking, dislodgement and pain at stoma site Most common GI side effects were constipation, nausea and diarrhoea
Halliday *et al.* (2017)	UK	Understand patients’ and carers’ experience of living with a jejunostomy feeding tube in the first months after surgery	Patient perspectives: HEN impacted sleep due to presence of tube, pain at stoma site or noise from pump Access to HEN team and support from dietitian around care of ET and stoma site was appreciated Support from, and knowledge of primary care providers around HEN viewed less positively
Bjuresäter *et al.* (2015)	Sweden	Understand the impact of HEN on daily life and how the situation can be managed	Patient perspectives: Necessary to provide verbal and written education on multiple occasions Education on daily care of ET, managing complications, and accessing support was important Those who struggled most did not feel sufficiently prepared and lacked support from HCPs Despite physical limitations and GI side effects of HEN, patients were grateful that HEN treatment meant survival and enabled them to stay at home
Madigan *et al.* (2007)	Northern Ireland	Explore GPs knowledge, attitudes and skills relating to enteral feeding in the community	GP perspectives: Perceived HEN as positive but had concerns about managing patients in a primary care setting Highlighted their lack of training on patients on HEN and problems that may arise Agreed training on HEN was needed and that such training should coincide with them having a patient on HEN rather than at random
Brotherton *et al.* (2007)	UK	Understand patients’ and carers’ experience of living with a PEG	Patient perspectives: HEN was time consuming, impacted sleep, and curtailed social activities but relieved pressure to consume a nutritionally adequate oral diet 20% of patients reported needing more support from HCPs
Jordan *et al.* (2006)	UK	Understand patients’ experience of living with a PEG	Patient perspectives: HEN impacted sleep and restricted participation in social activities Most common ET issues were leakage, dislodgement, and blockage Perceived that GPs and district nurses lacked knowledge Lack of knowledge of HCPs in ED increased burden of treatment for 4 patients Mean SF12 physical and mental health scores were below the average for the general population in the USA and below those for UK residents with chronic illness
Thompson *et al.* (2006)	USA	Understand patients’ experience of long-term HEN and how HCPs can support those on HEN	Patient perspectives: Physical limitations of HEN or underlying disease impacted daily activities Lack of support from HCPs resulted in patients attempting to resolve issues themselves 83% noted that inadequate HEN instruction led to confusion or fear around managing HEN Perceived that HCPs lacked expertise to address HEN-related problems Education and monitoring should include individualised care, discussing problems before they occur, and providing HEN education in stages
Liley *et al.* (2003)	UK	Understand patients’ and carers’ experience of HEN	Patient perspectives: Practical aspects of managing feed and equipment were inadequately covered during education All felt that HEN was worth undertaking and essential to survival Inexperience of some HCPs resulted in distress for some patients
L’Estrange (1997)	Northern Ireland	Understand patients’ and carers’ perspectives on HEN	Patient perspectives: Most noted that training had adequately prepared them for HEN Patients would benefit from more emphasis on managing ET issues, e.g., leakage, blockage 37% of patients were not satisfied with support from HCPs Patients expressed concern around lack of district nurse experience around HEN and stoma care
**PART B – Quantitative Studies**			
Martin *et al.* (2012)	Sweden	Investigate patients’ experience of living with a PEG and increase understanding of patients’ need for support	Patient perspectives: 73% of patients were satisfied with PEG and 82% did not feel limited in daily activities by PEG 60% did not find feeding too time consuming; however, this varied by age and education level Need for specialised and multidisciplinary care in managing HEN 80% of patients preferred to contact the PEG outpatient clinic, followed by home care team, then the dietitian, and primary care team
Brotherton *et al.* (2007)	UK	Compare the perceptions of patients, carers, nurses, and dietitians around home PEG feeding	Patient perspectives: 73% felt they received sufficient support from HCPs 13% stated that feeding regimen was not appropriate for home feeding 93% perceived HEN as successful and 80% believed QoL was acceptable HCP perspectives: 65% of dietitians and 83% of nurses believed that support from HCPs was sufficient 100% dietitians believed that feeding regimen was appropriate for home feeding Patients’ QoL viewed less positively by HCPs than by patients themselves
Paccagnella *et al.* (2007)		Assess the impact of HEN on QoL of patients and carers	Patient perspectives: HEN impacted autonomy in 43% of patients Advantages were physical well-being, less pressure to eat, hope for survival, and staying at home Mean values for psychological and physical functioning were relatively low Mean satisfaction score for social functionality was higher than psychological and physical scores
Loeser *et al.* (2003)	Germany	A prospective cross-sectional study with a longitudinal follow-up of 4 months to assess QoL in patients on HEN	Patient perspectives: When compared to EORTC reference data, functional scales were lower and symptom scales were higher at baseline Over 4 months, some aspects of QoL improved in both competent and non-competent patients EORTC scores increased for physical, emotional, and global functional scales but decreased for social functioning
McNamara *et al.* (2001)	Ireland	Assess the contribution of HCPs to the care of patients on HEN	HCP perspectives: 24% of GPs had ≥1 patient(s) on HEN under their care and 65% had experience of HEN GPs who attended nursing homes (77%) had significantly more exposure to tube feeding than those who did not Dietitians and nurses employed by nutrition companies noted inconsistent follow-up of the nutritional care needs of patients Almost all the company representatives felt that both GPs and PHNs need more education on EN
Roberge *et al.* (2000)	France	Evaluate the impact of HEN on QoL of life in patients treated for head and neck or oesophageal cancer	Patient perspectives: Global, physical and social functioning QLQ-C30 scores of QoL improved slightly between assessment at Day 7 of HEN and Day 28 HEN was responsible for not visiting family or close relations in 15% of patients and not going out in public in 23%
Schneider *et al.* (2000)	France	Assess QoL of patients on long-term HEN and evolution of QoL after initiation of HEN	Patient perspectives: EQ-5D and SF-36 scores of HEN patients were lower than reference values for age- and sex-matched general population All 38 patients felt that HEN had been at least ‘quite’ beneficial for them Mental well-being improved in 17 patients (15 due to HEN) and worsened in 7 patients (3 due to HEN) Physical well-being improved in 26 patients (25 due to HEN) and worsened in 1 patient (not due to HEN)

**Table 3.** Analysis of studies by theme

Author	Year	Importance of Initial Education	Impact of HEN and Complications on Daily Life	Need for Ongoing Support and Specialised Care	QOL of Patients on HEN
Ang *et al.*	2019	x		x	
Green *et al.*	2019	x	x	x	
Thomas *et al.*	2019		x	x	
Asiedu *et al.*	2018	x	x	x	
Halliday *et al.*	2017	x	x	x	
Bjuresäter *et al.*	2015	x	x	x	
Martin *et al.*	2012		x	x	
Brotherton *et al.*	2007			x	x
Madigan *et al.*	2007				
Paccagnella *et al.*	2007		x		x
Brotherton *et al.*	2007		x	x	x
Jordan *et al.*	2006		x	x	x
Thompson *et al.*	2006	x	x	x	
Liley *et al.*	2003	x	x	x	
Loeser *et al.*	2003				x
McNamara *et al.*	2001			x	
Roberge *et al.*	2000				x
Schneider *et al.*	2000				x
L’Estrange	1997	x		x	

### Importance of initial education

While patients reported receiving training on setting up and running the feed while in hospital, five studies noted that they would have liked further training on the practical aspects of HEN and caring for both the tube and stoma site (L’Estrange, 1997, Liley and Manthorpe, 2003, Thompson *et al.*, 2006, Asiedu *et al.*, 2018, Green *et al.*, 2019). Patients expressed the need for greater specificity and concreteness in educational material about how to manage HEN, e.g., complications and stoma care (L’Estrange, 1997, Asiedu *et al.*, 2018). Indeed, Bjuresäter *et al.* (Bjuresater *et al.*, 2015) found that the ability of service users to adapt to HEN was strongly related to the amount and quality of information and support they had received. Adaptation to HEN was facilitated by the provision of education on practical handling and daily care of the enteral tube (ET), management of complications, and where to seek support from HCPs.

### Impact of HEN and complications on daily life

Eight studies outlined how the feeding regimen, practical limitations, and complications of HEN impacted on the day-to-day life of patients. HEN disturbed mealtimes, sleep, daily activities, work, and travel, with one patient stating ‘we’re stuck here all day, can’t go far’ (Jordan *et al.*, 2006). Social activities outside the home were curtailed due to the time the feeding took and, in some cases, anxieties regarding the feeding tube being damaged (Thomas *et al.*, 2019). The most common tube-related issues were blockage, leakage, and dislodgement (Green *et al.*, 2019), while the most common GI complaints related to nausea, diarrhoea, and constipation (Jordan *et al.*, 2006, Asiedu *et al.*, 2018). Pain or infection at the stoma site was also noted in three studies (Halliday *et al.*, 2017, Asiedu *et al.*, 2018, Green *et al.*, 2019). Conversely, in a sample size of 104 adults with a PEG (Martin *et al.*, 2012), it was reported that 82% did not feel limited in daily activities by the PEG. Most patients (60%) did not find feeding too time-consuming; however, this varied by age and education level. Those with a university education were more likely to find feeding time-consuming and daily life disrupted by the PEG. Those over 65 years were also more likely to find feeding time-consuming.

Three studies reported that patients felt HEN was worth undertaking despite the limitations imposed on their life (Liley and Manthorpe, 2003), and they were grateful for the ability to stay at home (Bjuresater *et al.*, 2015). Indeed, some patients specifically expressed a preference for the management of HEN to be undertaken in their own home and actively avoided the hospital due to the time, discomfort, or prior experience, with one patient stating that ‘being at home is a hundred times better even if I’m still just as ill’ (Green *et al.*, 2019).

### Need for ongoing support and specialised care

The most common theme that emerged was the need for ongoing support from knowledgeable practitioners around the management of HEN and its complications. Access to support from HCPs was limited in many cases. Some patients reported having access to knowledgeable HCPs when an issue arose with the tube, while others described how a lack of access to a community HCP resulted in an acute admission to manage feeding challenge (Green *et al.*, 2019). Bjuresäter *et al.* found that the patients who struggled the most with HEN did not feel sufficiently prepared and lacked guidance and support from HCPs (Bjuresater *et al.*, 2015).

In a study (Halliday *et al.*, 2017) with a dedicated HEN team, the support from and accessibility to the HEN team were spoken about favourably by nearly all participants. Most participants noted that practical support from a HEN dietitian on care of the stoma site and tube was greatly appreciated. In contrast, support from primary care providers was viewed less favourably with one couple noting that the primary care provider ‘wasn’t really aware of jejs [jejunostomies] too much’. Five other studies indicated that patients lacked confidence in the knowledge of the HCPs they encountered when issues arose and expressed the need for non-specialist HCPs to improve their knowledge of HEN (L’Estrange, 1997, Liley and Manthorpe, 2003, Jordan *et al.*, 2006, Thompson *et al.*, 2006, Bjuresater *et al.*, 2015).

Two studies (McNamara *et al.*, 2001, Madigan *et al.*, 2007) assessed the experience of HCPs and HEN. Nurses and dietitians working within a company supplying HEN recognised the varying levels of experience with HEN in the community setting and highlighted the need for greater training in this area (McNamara *et al.*, 2001). In a survey of GPs, Madigan *et al.* (Madigan *et al.*, 2007) found that while almost half the sample of 23 GPs perceived HEN as a positive treatment for patients, others had serious concerns about the management of patients in the primary care setting. Lack of experience coupled with no training was highlighted as a problem. Some GPs felt that patients were discharged on EN without adequate consideration of the implications for the family and the patient, who were left unsupported in the community. Some doctors felt that because they did not know enough about HEN, their knowledge of the problems that may arise was also lacking.

### Quality of life (QoL) of patients on HEN

Five studies used specific indices to measure QoL in patients on HEN. In general, the scores indicate that patients on HEN have a poorer QoL than the general population, though it is difficult to separate this from their underlying disease.

A study on the impact of HEN on health-related quality of life (HRQoL) reported relatively low mean scores for psychological and physical functioning, as would be expected for patients with chronic illnesses and poor prognoses (Paccagnella *et al.*, 2007). However, the mean satisfaction score for social functionality was much higher (Paccagnella *et al.*, 2007). Similarly, Schneider *et al.* also found that HRQoL scores were lower than reference values for the age- and sex-matched general population; however, all 38 patients felt that HEN had been beneficial for them, with 63% stating it was ‘very’ beneficial (Schneider *et al.*, 2000). Conversely, other studies (Roberge *et al.*, 2000, Loeser *et al.*, 2003) found that QoL scores among adults on HEN improved slightly over the study period in terms of physical and global functional scales but decreased in terms of social functioning.

### Health economics of HEN

Table 4 summarises the findings of studies (*n*7) on the health economics of HEN. Six of the studies focused on the costs saved following introduction of a dedicated HEN service, while one study assessed complications experienced by HEN patients discharged to the community without structured follow-up. All studies used at least three metrics as a measure of the cost outcomes. Changes in the number of hospital admissions were an outcome of interest in all seven studies.

**Table 4.** Health economics of HEN

Author (Year)	Country	Sample Size	Costing Metrics Used	Cost Saved
Dinenage *et al.* (2015)	UK	*n*70	Estimated cost of enteral feed prescription and thickening agents for dysphagia for all patients^1^ Hospital admissions (frequency and bed days) for tube replacements and tube-related issues^2^ Hospital transport costs for tube replacements and tube-related issues^2^	For a cohort of 70 patients, the introduction of a HEN Team was associated with crude estimated cost savings of £111 272 over one year. The service cost £84 071 to deliver, giving rise to an estimated net saving of £27 201 to the NHS
		(*n*28 male)		£45 179 saved on enteral feed prescriptions per year^1^
				£1,278 saved on thickening agents for dysphagia^1^
				£64 341 saved on hospital admission^2^
				£474 saved on hospital transport costs^2^
Klek *et al.* (2014)	Poland	*n*314	Number of hospital admissions Length of hospital stay Costs of hospitalisation	Implementation of a specialised HEN care program significantly reduced the number of hospital admissions, average length of hospital stay, and mean annual costs of hospitalisation (*P* < .001)
		(*n*163 male)		Mean annual costs of hospitalisation (*n* = 314), US$
				Before HEN: 5513 ± 9043
				After HEN: 1619 ± 3592
				Number of hospital admissions (*n* = 312)
				Before HEN: 1.84 ± 2.4
				After HEN: 1.11 ± 2.1
				Average length of hospital stay (*n* = 312) in days
				Before HEN: 36.7 ± 74.8
				After HEN: 9.6 ± 19.4
Kurien *et al.* (2012)	UK	*n*313	Number of HEN team inputs Number of tube and stoma-related complications managed by HEN team Number of hospital admissions avoided	371 tube and stoma-related complications managed by HEN team.
		(*n*163 male)		227 hospital admissions avoided due to direct actions taken by HEN team.
				When compared with the historical cohort, there was a statistically significant reduction in readmission rates (2% vs 23%) for gastrostomy-related complications following the introduction of a dedicated enteral feed dietetic service.
Klek *et al.* (2011)	Poland	*n*313	Number of hospital admissions Length of hospital admissions Cost of hospital admissions	Implementation of a specialised HEN care program significantly reduced the number of hospital admissions (*P* < .001) as well as the length of hospital and ICU stays
		(*n*100 male)		Mean number hospital admissions (95% CI)
				Before HEN: 1.09 (0.96 – 1.22)
				After HEN: 0.21 (0.14 – 0.28)
				Mean duration of hospitalisation in days (95% CI)
				Before HEN: 20.84 (17.29 – 24.39)
				After HEN: 3.83 (2.13 – 5.53)
				Duration of ICU stay in days (95% CI)
				Before HEN: 2.35 (1.32 – 3.37)
				After HEN: 0.50 (0.09 – 0.92)
				Cost of hospitalisation, US$, per patient (95% CI)
				Before HEN: 764.65 (656.32 – 873.01)
				After HEN: 142.66 (85.02 – 199.72)
White *et al.* (2011)^3^	UK	*n*280	Number of HEN team inputs Number of hospital admissions avoided Number of replacement balloon gastrostomies	343 PEG-related complications seen by HEN team.
				103 patients required new balloon gastrostomies, of which 56 (43%) were performed as an emergency procedure by HEN dietitian.
				228 hospital admissions avoided due to direct actions taken by the HEN team.
White *et al.* (2008)^4^	UK	*n*180	Number of HEN team inputs Number of hospital admissions avoided Number of replacement balloon gastrostomies	545 PEG-related complications dealt with by HEN team.
				101 new balloon retained gastrostomies: 58 as emergency procedures (following PEG displacement, tube damage, or blockage) 43 planned, with no complications Hospital admissions were avoided in all 58 instances of PEG displacement, damage, or blockage by emergency replacement by the HEF dietitians
				69 patients admitted, only 15 (21%) were for PEG problems and all occurred at times of non-availability of staff at weekends or holidays or failure of carers to adhere to the written aftercare protocol.
Sanders *et al.* (2001)	UK	*n*87	Phone calls to endoscopy unit Number of home visits Number of PEG-related hospital admissions	During the 6-month study period, telephone advice was given 26 times with no further action required.
		(*n*42 male)		Home visits were necessary on 69 occasions.

**SD:** standard deviation.

**CI:** confidence interval.

1 : For total caseload of patients, *n* = 70.

2 : For caseload of 28 patients, based on number of admissions, bed days, and day cases.

3 : Published abstract in *Gut*.

4 : Published abstract in Proceedings of the Nutrition Society.

In a study that evaluated the impact of a dedicated HEN team (Dinenage *et al.*, 2015), the introduction of the team was associated with crude estimated cost savings of £111 272 over one year. The service cost £84 071 to deliver, giving rise to an estimated net saving of £27 201 to the NHS. The study also measured patient satisfaction and all respondents measured the service as good or excellent.

Three studies in the UK found that introduction of a HEN team was associated with a reduction in hospital admission rates due to HEN-related complications. One study (Kurien *et al.*, 2012) reported a statistically significant reduction in readmission rates (2% vs 23%) for gastrostomy-related complications following the introduction of a dedicated enteral feed dietetic service, when compared with a historical cohort. Another (White *et al.*, 2011) found that 343 PEG-related complications were handled by the HEN team during a one-year period, and 228 hospital admissions were avoided due to direct actions taken by the HEN team. Finally, 545 PEG-related complications were dealt with by the HEN team, and hospital admissions were avoided in all fifty-eight instances of PEG displacement, damage, or blockage by emergency replacement by the HEN dietitians (White *et al.*, 2008).

In Poland, two multi-centre studies found that implementation of a specialised HEN service significantly reduced the number of hospital admissions, length of stays and costs of hospitalisation (Klek *et al.*, 2011, Klek *et al.*, 2014). It must be noted, however, that prior to the implementation of the HEN service, patients on HEN were using homemade, rather than commercial formula.

A 6-month prospective analysis of the support required by 87 patients discharged with a PEG found that telephone advice was given on 26 occasions with no further action needed. Sixty-nine home visits were required with the main indications being issues with the stoma site, e.g., over-granulation, or tube-related issues, e.g., blockage or dislodgement (Sanders *et al.*, 2001).

## Discussion

This scoping review makes an important contribution to the argument for specialist HEN care in the primary care setting. The most common theme to emerge from the literature was that service users want and need ongoing support from knowledgeable HCPs. The literature also highlighted how such a service can save costs, reduce unnecessary hospital admissions, and improve quality of care and service user satisfaction (Dinenage *et al.*, 2015).

Outcomes are optimised when HEN teams, rather than single providers, manage this diverse patient population (Johnson *et al.*, 2019). The National Institute for Health and Care Excellence (NICE) (National Institute for Health and Care Excellence, 2006) and ESPEN (Bischoff *et al.*, 2020) guidelines on HEN outline the importance of coordinated, multidisciplinary care. The team should consist of the GP, public health nurse, community pharmacist, dietitian, and other allied health professionals, e.g., speech and language therapists, as appropriate. However, it may be most realistic for community dietitians to provide specialist care for patients on HEN. A HEN dietitian is ideally placed to upskill in stoma care and tube management (Stanley and Borthwick, 2013). GPs and PHNs may have insufficient numbers on individual caseloads to maintain the necessary expertise and competency (Liley and Manthorpe, 2003). While support from knowledgeable HCPs, e.g., a HEN dietitian, was viewed positively (Halliday *et al.*, 2017), patients felt that non-specialist HCPs in the community lacked the expertise to address HEN-related problems (L’Estrange, 1997, Liley and Manthorpe, 2003, Jordan *et al.*, 2006, Thompson *et al.*, 2006, Bjuresater *et al.*, 2015).

While underlying disease may negatively impact patients’ QoL, if day-to-day HEN is not working well, this will undoubtedly negatively impact patients’ QoL. The literature highlighted the importance of initial education and training to help patients adapt to HEN. Anxiety and illness in hospital may prevent patients from fully grasping EN, and follow-up education within the home environment is essential (Madigan, 2003). Difficulties may only arise post discharge, and it is critical that patients have access to knowledgeable HCPs who can answer their questions. Patients frequently expressed dissatisfaction at the level of knowledge of HCPs encountered when issues arose. Indeed, in a study by Jordan *et al.* (Jordan *et al.*, 2006), lack of knowledge of PEG tubes in the emergency department increased the burden of treatment for four patients. Furthermore, in a survey of GPs, Madigan *et al.* (Madigan *et al.*, 2007) found that GPs themselves noted that training on caring for patients on HEN was ‘non-existent’ and that HEN was ‘something that has just landed with us’.

The literature supports the economic benefit of a HEN service. A specialist HEN service can manage complications and reduce unnecessary hospital admissions. Adequate education of all HCPs involved in the care of patients on HEN will optimise the capacity of patients to live well at home. Furthermore, a dedicated service can facilitate the delivery of quality care in the less costly primary care environment. It is reasonable to suggest that significant healthcare savings could be achieved if this service gap was addressed.

The measures used to estimate the cost savings of a HEN service varied from actual costs saved by having such service, to the number of HEN team inputs and reductions in hospital admissions. Many common tube-related issues such as blockages or dislodgement may be dealt with at a lesser cost by having a specialist service. Furthermore, early recognition and treatment of non-urgent complications can save costs and hospital admissions (White *et al.*, 2008). In addition to providing practical support around the feeding regimen and ET, a HEN dietitian may provide reassurance for patients, particularly in the initial stages of adaptation to HEN. Adequate follow-up and early intervention around complications will also enable patients to remain in their home environment and attenuate negative impacts on QoL.

The authors acknowledge the limitations of this review. There are limitations to scoping review methodology, as the focus is on providing breadth rather than depth of information on a particular topic. The studies did not undergo a quality appraisal as this is outside the typical scope of scoping reviews. For example, while a systematic review generally focuses on a relatively narrow range of quality-assessed studies (Arksey and O’Malley, 2005), a scoping review serves to provide a complete overview of all relevant literature related to a topic (Levac *et al.*, 2010). As the literature on HEN expands, quality appraisals of research on specific aspects of HEN provision will be valuable to inform high-quality evidence-informed practice. While the search strategy was devised with assistance of a medical librarian, all relevant literature may not have been identified given resource constraints. Finally, the experience of carers was not included in this scoping review. Given that carers may play a central role in HEN provision and support, future research on their perspective could provide useful insight to improve the service delivery of HEN. Advantages of the methodology include the consistent use of the Arksey and O’Malley framework throughout the process. To ensure a broad search of the literature, the comprehensive search strategy included four electronic databases. Additionally, experienced stakeholders were consulted at regular intervals to facilitate the appropriate identification of themes from the literature.

Although the value of HEN to service user outcomes is clear, gaps remain in the knowledge of HCPs and in our understanding of the economics of HEN. A multi-centre prospective study comparing standardised health and economic outcomes, such as number of ET-related hospital admissions between areas with and without specialist HEN services, would provide useful data on the relative merit of a HEN service. While the studies included in this scoping review identified the need for specialised care from HCPs to support HEN, future studies should specifically explore the extent and scope of the dietitian’s role in providing that expertise and competency. Furthermore, future research could also investigate if training of community HCPs by HEN dietitians leads to a reduction in costs and hospital admissions, e.g., through earlier detection of, and intervention around, complications.

Additionally, the studies on the experience of HCPs took place in 2001 (McNamara *et al.*, 2001) and 2007 (Brotherton *et al.*, 2007, Madigan *et al.*, 2007). Future research could identify whether the views of HCPs have changed in the intervening period. As the prevalence of HEN has grown (Ojo, 2015), HCPs today may have more knowledge and experience on management of HEN in the primary care setting.

While service users on HEN may have complex underlying problems, many encounter issues specific to the enteral tube (Kurien *et al.*, 2012). A specialist HEN service can manage complications and reduce unnecessary hospital admissions. Such a service can support service users to live well at home and support the health system to reorient service delivery towards the primary care setting in line with current and anticipated trends in healthcare.

## Supplementary material

For supplementary material accompanying this paper visit http://doi.org/10.1017/S1463423622000366. click here to view supplementary material
