# Supplementary material for: A scoping review of best practices in home enteral tube feeding
Source: Prim Health Care Res Dev. 2022 Aug 4;23:e43. doi: 10.1017/S1463423622000366 (PMC9381166; doi:10.1017/S1463423622000366)
Supplement: Supplementary file 1 [file phcsup.zip › S1463423622000366sup001.docx]

## **Appendix I – Search Strategy**

| **Database** | **Keywords** |
| --- | --- |
| EMBASE | 'enteric feeding'/exp OR 'home enteral nutrition'/exp  ((Enteral OR enteric) NEAR/2 (feeding OR nutrition)):ti,ab  ('intragastric feeding' OR 'intestinal feeding' OR 'tube feeding'):ti,ab  'gastrostomy'/exp  gastrostom*:ti,ab  'percutaneous endoscopic gastrostomy'/exp  ('percutaneous endoscopic gastrostomy' OR PEG):ti,ab  #1 OR #2 OR #3 OR #4 OR #5 OR #6 OR #7  'home'/exp OR 'home care'/exp OR 'community care'/exp OR 'community'/exp OR 'community hospital'/exp  ((Enteral OR enteric) NEAR/2 (feeding OR nutrition) NEAR/4 (home* OR communit*)):ti,ab  ((gastrostom* OR 'intragastric feeding' OR 'intestinal feeding' OR 'tube feeding' OR 'percutaneous endoscopic gastrostomy' OR PEG) NEAR/4 (home* OR communit*)):ti,ab  #9 OR #10 OR #11  #8 AND #12 |

| **Database** | **Keywords** |
| --- | --- |
| Web of Science | TS =((((Enteral OR enteric) NEAR/2 (feeding OR nutrition)) OR “intragastric feeding” OR “intestinal feeding” OR “tube feeding” OR gastrostom* OR “percutaneous endoscopic gastrostomy” OR PEG) AND (((Enteral OR enteric) NEAR/2 (feeding OR nutrition) NEAR/4 (home* OR communit*)) OR ((gastrostom* OR “intragastric feeding” OR “intestinal feeding” OR “tube feeding” OR “percutaneous endoscopic gastrostomy” OR PEG) NEAR/4 (home* OR communit*)))) |

| **Database** | **Keywords** |
| --- | --- |
| Medline | Enteral Nutrition/  ((Enteral OR enteric) adj2 (feeding OR nutrition)).ti,ab.  (intragastric feeding OR intestinal feeding OR tube feeding).ti,ab.  gastrostomy/  gastrostom*.ti,ab.  (percutaneous endoscopic gastrostomy OR PEG).ti,ab.  or/1-6  exp Community Health Services/  ((Enteral OR enteric) adj2 (feeding OR nutrition) adj4 (home* OR communit*)).ti,ab.  ((gastrostom* OR intragastric feeding OR intestinal feeding OR tube feeding OR percutaneous endoscopic gastrostomy OR PEG) adj4 (home* OR communit*)).ti,ab.  or/8-10  7 AND 11 |

| **Database** | **Keywords** |
| --- | --- |
| CINAHL | (MH "Enteral Nutrition")  TI ((Enteral OR enteric) N2 (feeding OR nutrition)) OR AB ((Enteral OR enteric) N2 (feeding OR nutrition))  TI (“intragastric feeding” OR “intestinal feeding” OR “tube feeding”) OR AB (“intragastric feeding” OR “intestinal feeding” OR “tube feeding”)  (MH "Gastrectomy+")  TI (gastrostom*) OR AB (gastrostom*)  TI (“percutaneous endoscopic gastrostomy” OR PEG) OR AB (“percutaneous endoscopic gastrostomy” OR PEG)  #1 OR #2 OR #3 OR #4 OR #5 OR #6  (MH "Home Health Care") OR (MH "Home Nutritional Support") OR (MH "Home Rehabilitation") OR (MH "Home Health Aides") OR (MH "Rehabilitation, Community-Based") OR MH "Community Health Centers+") OR (MH "Community Networks") OR (MH "Community Role") OR (MH "Communities+") OR (MH "Community Living+"))  TI ((Enteral OR enteric) N2 (feeding OR nutrition) N4 (home* OR communit*)) OR AB ((Enteral OR enteric) N2 (feeding OR nutrition) N4 (home* OR communit*))  TI ((gastrostom* OR “intragastric feeding” OR “intestinal feeding” OR “tube feeding” OR “percutaneous endoscopic gastrostomy” OR PEG) N4 (home* OR communit*)) OR AB ((gastrostom* OR “intragastric feeding” OR “intestinal feeding” OR “tube feeding” OR “percutaneous endoscopic gastrostomy” OR PEG) N4 (home* OR communit*))  #9 OR #10 OR #11  #8 AND #12 |
