# Supplementary material for: A scoping review of best practices in home enteral tube feeding
Source: Prim Health Care Res Dev. 2022 Aug 4;23:e43. doi: 10.1017/S1463423622000366 (PMC9381166; doi:10.1017/S1463423622000366)
Supplement: Supplementary file 1 [file phcsup.zip › S1463423622000366sup002.docx]

## **Supplementary material – Data Extraction Tables**

## **Patient and HCP Experiences of HEN**

| **Author (Year)** | **Country** | **Study Aim** | **Key Findings** | **Notable Quote** |
| --- | --- | --- | --- | --- |
| **PART A – Qualitative Studies** | | | |  |
| Ang et al  (2019) | Singapore | Understand patients’ and carers’ experience upon initiation of long-term HEN | (IE): A lack of adequate knowledge on the procedure of PEG insertion and possible complications increases the anxiety level and fear of patients towards the procedure of PEG insertion. |  |
|  |  |  | (NS) Patients appreciated accessibility and support of nutrition nurse to provide advice as well as technical support for complications e.g. tube breakage or dislodgement. |  |
| Green et al  (2019) | UK | Understand patients’ and carers’ experience of long-term HEN | Almost all participants stated that they preferred management of ET-related issues to be undertaken in their own home. | “Being at home is a hundred times better even if I’m still just as ill” |
|  |  |  | (IE) Many participants reported receiving some training in managing the tube in hospital prior to discharge, although some would have liked more opportunity to  learn the procedures required to care for the tube. This lead to feelings of anxiety on discharge. | “You feel at a loss to begin with, and it’s a bit worrying for family as well.” |
|  |  |  | (IC) All participants described the impact of HEN on day-to-day life, requiring significant changes to activities of daily living and how participants planned holidays and  managed work.  A range of tube related problems related were reported including dislodgement,  stoma infection and over-granulating tissue. |  |
|  |  |  | (NS) Support from HCPs was described as very limited by some participants, with little contact with HCPs experienced in the management of EN.  Some reported knowing and having access to knowledgeable HCPs when an issue arose with the tube though others described how their inability to contact a community HCP able to provide support resulted in admission. | “…if you’ve got a problem you don’t really know who to speak to because you  don’t see these on a regular basis”  “you go to Accident and Emergency and I don’t think they are always ready and able to look after a PEG that has fallen out” |
| Thomas et al  (2019) | UK | Establish the impact of HEN on daily life of those with a diagnosis of head and neck cancer | (IC) Participants felt out of control of their daily lives secondary to restrictions imposed by the feeding regime. HEN disturbed meal-times, sleep, daily activities, work and travel. Social activities outside the home were curtailed as a result of the time tube feeding took and anxieties regarding the feeding tube being damaged. | "my total life is like taken over by this pump feeding at night" |
|  |  |  | (NS) Regaining independence and control facilitated adaptation to HEN. Empowerment was achieved through knowledge and skill development, increasing ﬂexibility in the feeding regime and minimising side-effects related to the feed. | Rather than the district nurse, they said, we’ll be here between 8 and 5, you were stuck in the house. But we’d rather do it ourselves, then you can manage your time better |
| Asieudu et al  (2018) | USA | Understand patients’ and carers’ experience of long-term HEN | (IE) Early education delivered by the HEN team regarding tube feeding and its benefits reduced fear and resistance to HEN however, patients also expressed the need for greater specificity and concreteness in educational material about what to expect with HEN e.g. complications. |  |
|  |  |  | (IC) Patients noted both mechanical and physical complications of the stoma site, such as a leaking tube or the tube falling out and pain and discomfort at the stoma site.  GI side effects such as bloating, constipation, nausea and diarrhoea also reported. |  |
|  |  |  | (NS) Although patients acknowledged having received comprehensive information about HEN, they identified a need for ongoing education regarding the practical logistics of doing HEN. |  |
| Halliday et al  (2017) | UK | Understand patients’ and carers’ experience of living with a JFT in the first months after surgery | (IE) As part of routine practice, all patients in this study were trained by a registered dietitian before discharge from the hospital and provided with detailed written information and a 24/7 support line from the homecare company provided. |  |
|  |  |  | (IC) HEN impacted on ability to sleep either due to presence of tube, stoma site or noise from pump if fed overnight. Patients reported anxiety over tube falling out, especially if fed overnight. The stoma site was the source of physical problems for patients, describing it as “swollen”, “sore”, “leaking”, and “infected”. |  |
|  |  |  | (NS) Support from and accessibility of HEN team was spoken about favourably from nearly all participants. Most patients noted that practical support from HEN dietitian around stoma site and tube care was greatly appreciated. In contrast, support from primary care providers was viewed less favourably with one couple noting that the PCP “wasn’t really aware of jejs too much”. |  |
| Bjuresäter et al (2015) | Sweden | Understand the impact of HEN on daily life and how the situation can be managed | (IE) The patients’ ability to adapt to HEN was strongly related to the amount and quality of information and support they had received. When they expressed that pre-discharge information and instructions were satisfactory, they had received information about practical handling and daily care of the tube feeding, how to deal with complications and where to seek support from HCPs.  Furthermore, the information was given repeatedly both orally and in written form. |  |
|  |  |  | (IC) Patients were grateful for the fact that HEN treatment meant survival and gave them the opportunity to stay at home. However, the patients experienced reduced capacity due to impaired physical functions and side effects, such as early satiety, nausea and diarrhoea and the time-consuming nature of HEN which reduced ability to carry out daily activities. | I could not increase the speed even a little bit, because then I became nauseous. |
|  |  |  | (NS): All patients expressed a need for appropriate guidance and support but the amount and quality of support from HCPs varied from patient to patient.  The patients who struggled most with HEN did not feel sufficiently prepared and lacked guidance and support from health care personnel. They were uncertain about how to handle certain issues in their treatment, and how to act when problems occurred. | I did get some information at the hospital, it’s true, but the information shouldn’t be given too quickly.  It was too much all at once so I was very confused ... but I thought I would manage things when I  came home, but I didn’t ... it was too much. It is so overwhelming, and so much to keep track of. |
| Madigan et al  ^(^2007) | Northern Ireland | Explore GPs knowledge, attitudes and skills relating to enteral feeding in the community | **Previous experience of EN:** Most mentioned that their main experiences of tube feeding were when they were working in the hospital environment, which, for many, was some years previously ***Attitude to HEN:** Just under half the sample perceived HETF as a positive treatment for patients but others had serious concerns about the management of patients in the primary care setting **#Key issues:** Lack of experience coupled with no training was highlighted as a problem for some. Some doctors felt that because they did not know enough about the treatment their knowledge of the problems that may arise was also lacking. Almost every respondent mentioned that they did need some basic training in the area.  **-Ideal training:** Approximately half of the respondents felt that the most effective training would be delivered locally and, more specifically, within the practice. Most wanted it to be short. The internet, handouts and a small booklet were mentioned, as was a simulation course. Others wanted basic information, the chance to see the tubes and equipment and to ask questions from the facilitator. Most did not care who the facilitator was, "so long as they knew something about it".. GPs also felt that training is much more appropriate when they have a patient rather than having random training sessions. | "enteral tube feeding was mostly when I was in hospital all those many years ago. PEG feeding well, I have had a couple of patients with it but I wouldn't say I knew an awful lot about it"  *: I think it is important and it has its place but I think that currently there is no one taking control about the initiation of it and the reasons for initiating it. There would be a lack of understanding about it in the primary care community" #:"there has been a problem with the PEG tube and I have been asked to sort something out, I feel I don't really have much experience to do that" "The training has been non existent. This is something that has just landed with us." "I don't know enough about them to know what difficulties there are because I have only had one patient and they had no problems" "We need to know something about it for two reasons, one to be able to provide a service and secondly people expect us to know something about it and if we are sounding rather lacking in knowledge it doesn't do very much for the confidence level"  - "You could be very much up to scratch now and because you have no practice with it in five years time you are back to square one" |
| Brotherton et al  (2006) | UK | Understand patients’ and carers’ experience of living with a PEG | (IC) Although the majority of patients were happy with the feeding regimen, the feeding itself was time-consuming and impacted ability to participate in social activities.  Patients reported disturbed sleep due to overnight feeding and the presence of PEG tube.  On the positive side, patients noted that PEG feeding relieving pressure at mealtimes as there was no longer the need to consume a nutritionally adequate diet orally. | ‘It restricts me from going out’ and ‘If we go on holiday that is a nightmare; just the sheer volume of feed that we have to take.’  ‘It disturbs my sleep so much. When you go to bed and this tube is just sticking out of your stomach, you cannot move.’  ‘It has taken the pressure off at mealtimes; I was finding it very difficult to eat. I was spending hours on each meal and not finishing it’ |
|  |  |  | (NS) 11 of the 15 patients reported receiving sufficient support from HCPs however 3 of the 15 (20%) reported needing more support than currently receiving. |  |
|  |  |  | (QOL): 12 of the 15 patients reported having an acceptable QoL, 1 unsure, 2 No. The two patients who reported their QoL was not acceptable reported that this was not due to the PEG, but the underlying disease. |  |
| Jordan, et al (2006) | UK | Understand patients’ experience of living with a PEG | (IC) The feeding regimen and practical difficulties of PEG limited participants and entailed the loss of important social aspects of their lives.  The most common complications reported included nausea, tube leakage, dislodgment and blockage and disturbed sleep due noise from pump. | ‘We’re stuck here all day, can’t go far.’ |
|  |  |  | (NS) Thirteen of the 20 participants commented that HCPs including district nurses, general practitioners needed more knowledge of PEG feeding. This was contributing to patients’ feelings of isolation. Lack of knowledge of PEG tubes in accident and emergency departments increased the burden of treatment for four patients.  Six participants felt that it would be useful to have one named (knowledgeable) person whom they could telephone if they  had problems. | ‘The nurses still haven’t been told how to treat it, to move the PEG in and out, to make sure it doesn’t stick inside my stomach, causing ulcers. We’ve been given a leaflet, but they haven’t been sent it.’ |
|  |  |  | (QOL): The mean SF12 physical and mental health scores were below the average for the general population in the USA and below those for UK residents with chronic illness.  Disentangling the impact of the PEG from the underlying illness was difficult, but one participant with a lifetime’s experience of illness estimated that current PEG technology  was responsible for some loss of quality of life. | ‘I’d say it’s about a 20% deterioration of your life, just the PEG.’ |
| Thompson et al (2006) | USA | Understand patients’ experience of long-term HEN and how HCPs can support those on HEN | (IC): The majority of patients experienced physical limitations due to HEN itself or underlying disease and were required to alter activities or accept assistance from others. Many altered their prescribed HEN regimen to meet their lifestyle or minimize the disruption e.g., altering feeding schedule.  All patients worked to resolve their own problems e.g., seeking information, searching for the right equipment or adapting what was available. The need to be self-taught often stemmed from a lack of adequate instruction, resources, or support. | “There’s no help. You have to  kind of ‘self-help’ yourself on the situation.” |
|  |  |  | (NS) All 12 participants emphasised the importance of support from HCPs. While four participants with protracted HEN-related problems (e.g., skin-care issues and weight loss) discussed the value of a close relationship with their HCP, 8 patients did not think their HCP improved their ability to adapt to or cope with HEN. Ten participants complained of inadequate HEN instruction and reported experiencing confusion, frustration, or fear of the unknown because they felt unprepared to assume the responsibility of their HEN regimen or to deal with complications such as tube dislodgement.  Five participants relied on their primary HCP or wound care specialist for answers, yet others perceived that their HCP lacked the expertise to address HEN related problems.  Participants offered suggestions for HCPs to improve the HEN education and monitoring processes e.g., individualised care, discussing problems before the occur, providing HEN education in stages, etc. A focus group of healthcare professionals validated the participants’ concerns and acknowledged the feasibility of implementing many practice changes at the clinician level. | “I don’t think I felt the anger so much until I  realized that I hadn’t had much help from the healthcare industry.” |
| Liley et al  (2003) | UK | Understand patients’ and carers’ experience of HEN | (IE): Some patients reported that the practical aspects of managing feed and equipment had been inadequately covered in their preparation. While techniques were reported as being demonstrated and taught, there was a lack of support in integrating the process of HEN into other household routines and spaces. |  |
|  |  |  | (IC): There was uniform recognition that, despite all its problems, HEN was effective and worth undertaking. All patients felt that HEN was essential to survival. Portraying HEN as a normal part of daily living made it possible for patients to feel that they could enjoy other elements of ordinary life. |  |
|  |  |  | (NS): This rapid building up of expertise enabled patients to recognise the inexperience of some HCPs of the health professionals whom they encountered. Some distress was reported when HCPs professionals did not meet patients’ standards. Lack of confidence in practitioners influenced how patients engaged with professionals, shifting from dependency to independence rapidly. |  |
| L’Estrange (1997) | Northern Ireland | Understand patients’ and carers’ perspectives on HEN | (IE): All 19 patients living at home felt they had sufficient time in hospital to prepare for HEN (mean time 2 weeks) and 15 felt satisfied that their training had adequately prepared them for HEN. The benefits of being able to change the feed when still in hospital and of the literature received were highlighted. Patients felt that more emphasis should have been placed on the causes of pump alarming, preventing leaks, how to run feed properly through the giving set, preventing and treating tube blockages, and on stoma care. |  |
|  |  |  | (NS): Whilst 12 of the 19 patients at home expressed satisfaction with the level of support received since coming home, seven were not satisfied. The issues of concern included: not being weighed regularly, lack of district nurse experience with HEN, stoma care and lack of emotional support for not being able to eat. District nurse contact was regularly occurring for 16 patients, ranging from daily to weekly visits, whilst three patients no longer had regular contact. |  |
| **PART B – Quantitative Studies** | | | | |
| Martin et al (2012) | Sweden | Investigate patients’ experience of living with a PEG and increase understanding of patients’ need for support | (IC): The majority (73%) of patients were satisfied with the PEG. The majority (82%) of patients did not feel limited in daily activities by PEG and most (60%) did not find feeding too time consuming however this varied by age and education level. Those with a university education were more likely to find feeding time consuming and daily life disrupted by the PEG while those over 65 years were more likely to find feeding time consuming. |  |
|  |  |  | (NS): The need for specialised and multidisciplinary care in managing PEGs at home was highlighted. 83 of the 104 patients preferred to contact the PEG outpatient clinic with questions or problems around the PEG itself and feeding, with 15 contacting their home care team, 13 the dietitian, 9 contacting the primary care team. |  |
| Brotherton et al (2007) | UK | Compare the perceptions of patients, carers, nurses, and dietitians around home PEG feeding | (NS): Many of the patients were not in regular contact with their GP; district nurses often provided care and the involvement of the GP was only sought when necessary. The majority (73%) of patients felt they received sufficient support from HCPs though just 65% of dietitians and 83% of nurses believed it to be sufficient.  While 100% of dietitians believed that the feeding regimen was appropriate for home feeding, not all patients agreed, with 13% stating it was not appropriate. |  |
|  |  |  | (QOL) The majority (93%) of patients perceived the PEG feeding as being successful and most (80%) believed they had an acceptable QoL, however patients themselves viewed their QoL more positively than did both carers and HCPs. If given the choice, the majority (53%) of patients would stop the feeding though just 20% dietitians and 6% nurses would do same. |  |
| Paccagnella et al (2007) |  | Assess the impact of HEN on QoL of patients and carers | (IC): 43% of patients reported that HEN impacted autonomy. Others reported disadvantages of HEN included oral side effects from lack of food and drinks and GI side effects such as constipation.  The main advantages of HEN for patients included physical wellbeing, relieving the pressure to eat, hope for survival and the ability to stay at home. | "I can't move"  "I have to stay always at home"  ‘without, I couldn’t live’  "I can stay with my wife" |
|  |  |  | QOL: The results of the SAT-P test indicated that mean scores for psychological and physical functioning are relatively low, as expected for patients with chronic illnesses with poor prognosis. However, the mean satisfaction score for social functionality is much higher. |  |
| Loeser et al (2003) | Germany | A prospective cross-sectional study with a longitudinal follow-up of 4 months to assess QoL in patients on HEN | QOL: When compared with the EORTC reference data, functional scales were lower and symptom scales were higher in HEN patients. QOL was significantly reduced in non-competent patients which was obvious for all dimensions of the Spitzer and Karnofsky indices (activity, daily living, health, support, outlook).  Study 2 (longitudinal over 4 months):  In a longitudinal analysis over 4 months, some aspects of QoL improved. The Spitzer and Karnofsky indices improved in competent and non-competent patients, however, a lower QOL was observed in non-competent than in competent patients. EORTC scores (measured in competent patients only) increased in terms of physical, emotional and global functional scales but decreased in terms of social functioning. |  |
| McNamara et al (2001) | Ireland | Assess the contribution of HCPs to the care of patients on HEN | **GPs:** 24% of GPs had one or more enterally tube-fed patients in their care at that time, with 65% reporting having had such a patient in their care in the past. GPs who attended nursing homes (77% of the responders) had significantly more exposure to tube feeding than those who did not. None of the GPs who did not attend nursing homes had adult patients on enteral tube feeding in their care at the time of the survey.  **Nutrition company employees:**  Ten company employees (seven dietitians and three nurses) from six different nutrition product companies were interviewed. Representatives described their roles as `educator', `support person', `co-ordinator', `liaison', `nutritional advisor', `facilitator' and `after sales service provider'. The question of inconsistent follow-up of the nutritional care needs of these patients was an area of concern to the company representatives, many of whom described the dilemma facing them when encountering patients experiencing difficulties with their tube feeds. All of the company representatives recognized that it is inappropriate for them to be involved in nutritional management decisions but stated that they are frequently asked to deal with such issues. Participants believed that the process of discharging and managing patients on HETF needed to be more structured and better co-ordinated with universal protocols and particular individuals (preferably community dietitians) responsible for the nutritional care of patients in the community. Almost all of the company representatives felt that both GPs and PHNs need more education on enteral nutrition, and regarded this as a priority in the improvement of HETF.  Key finding:  Many of the problems faced by patients outlined by the company representatives in this survey (e.g. tube blockages, leaking PEGs), could be dealt with in the community at lesser cost if existing personnel (e.g.PHNs, GPs) were appropriately trained or if additional personnel with specialist experience were available (e.g. dietitians). In view of the considerable difficulty involved in educating a large number of GPs and PHNs to an appropriate level to deal with relatively few patients each, it would seem more reasonable to consider the need for community dietitians in providing this increasingly needed service. |  |
| Roberge et al (2000) | France | Evaluate the impact of HEN on QoL of life in patients treated for head and neck or oesophageal cancer | QOL: Global, physical and social functioning QLQ-C30 scores of QoL improved slightly between assessment at Day 7 of HEN and Day 28 however HEN was responsible for not visiting family or close relations in 15% of patients, and not going out in public in 23%. |  |
| Schneider et al (2000) | France | Assess QoL of patients on long-term HEN and evolution of QoL after initiation of HEN | QOL: EQ-5D and SF-36 scores of HEN patients were lower than reference values. The differences were all statistically significant except for body pain, vitality, and mental health however, a trend towards significance was observed for these last variables.  However, all 38 patients felt that HEN had been very (n = 24), rather (n = 12) or quite (n = 2) beneficial for them. Mental well-being had improved in 17 patients (15 due to HEN), had remained stable in 14 patients (five due to HEN), and had worsened in seven patients (three due to HEN). Changes were more dramatic concerning physical well-being, which had improved in 26 patients (25 due to HEN), was stable in 11 (nine due to HEN) and had worsened in one patient (not due to HEN |  |

**Key Themes**

IE – Importance of initial education

IC – Impact of HEN and common complications on daily life

NS – Need for ongoing support and specialised care

QOL – Quality of life scores

## **Health Economics of HEN**

| **Author (Year)** | **Country** | **Sample Size** | **Costing Metrics Used** | **Costs Saved** | **Notes** |
| --- | --- | --- | --- | --- | --- |
| Dinenage et al (2015) | UK | *n*70  (*n*28 male) | Estimated cost of enteral feed prescription and thickening agents for dysphagia for all patients  Frequency and length of hospital admission and hospital transport costs for ETF related problems for 28 patients in the caseload. | For a cohort of 70 patients, the introduction of a HEN Team was associated with crude estimated cost savings of £111,272 over one year. The service cost £84,071 to deliver, giving rise to an estimated net saving of £27,201 to the NHS  £45,179 saved on enteral feed prescriptions per year^1^  £1,278 saved on thickening agents for dysphagia^1^  £64,341 saved on hospital admission^2^  £474 saved on hospital transport costs^2^ | HEN team = dietitian, speech and language therapist, homecare company nurse and community nurse. Aim of team = improve patient experience and quality of nutritional care.  Pre-post evaluation of impact of HEN team undertaken.  Also measured patient satisfaction:  Survey returned by *n* = 14 with 100% of respondents rating service as good or excellent |
| Klek et al (2014) | Poland | *n*314  (*n*163 male) | Number of hospital admissions  Length of hospital stay  Costs of hospitalisation | Mean annual costs of hospitalisation in adults (*n* = 314), US$  Before HEN: 5513 ± 9043  After HEN: 1619 ± 3592  Hospital admissions in adults (*n* = 312)  Before HEN: 1.84 ± 2.4  After HEN: 1.11 ± 2.1  Average length of hospital stay in adults (*n* = 312) in days  Before HEN: 36.7 ± 74.8  After HEN: 9.6 ± 19.4  (P < 0.001) | Two 12-mo periods were compared. During the first period, patients were tube fed a homemade diet and were not monitored; during the other period, patients received HEN. HEN included tube feeding and complex monitoring by a nutrition support team.  EN significantly reduced the number of hospital admissions and the length of hospital stay. The need for hospitalization was significantly reduced. |
| Kurien et al (2012) | UK | *n*313  (*n*163 male) | Number of HEN team inputs  Number of tube and stoma-related complications managed by HEN team  Number of hospital admissions avoided | 371 tube and stoma-related complications managed by HEN team.  227 hospital admissions avoided due to direct actions taken by HEN team.  When compared with the historical cohort, there is a statistically significant reduction in readmission rates (2% vs 23%) for gastrostomy related complications following the introduction of a dedicated enteral feed dietetic service | HEN team = two dietitians and a dietetic assistant |
| Klek et al (2011) | Poland | *n*313  (*n*100 male) | Number, length, and cost of hospital admissions | Implementation of a specialized HETF care program significantly reduced the number of hospital admissions as well as the length of hospital and ICU stays (Table 2)  Mean number hospital admissions (95% CI)  Before HEN: 1.09 (0.96 – 1.22)  After HEN: 0.21 (0.14 – 0.28)  Mean duration of hospitalisation in days (95% CI)  Before HEN: 20.84 (17.29 – 24.39)  After HEN: 3.83 (2.13 – 5.53)  Duration of ICU stay in days (95% CI)  Before HEN: 2.35 (1.32 – 3.37)  After HEN: 0.50 (0.09 – 0.92)  Cost of hospitalisation, US$, per patient (95% CI)  Before HEN: 764.65 (656.32 – 873.01)  After HEN: 142.66 (85.02 – 199.72)  P < .001 | Two 12-mo periods were compared. During the first period, patients were tube fed a homemade diet and were not monitored; during the other period, patients received HEN. HEN included tube feeding and complex monitoring by a nutrition support team.  Implementation of a specialized HETF care program significantly reduced the number of hospital admissions as well as the length of hospital and ICU stays.  The need for hospitalization and ICU admission was significantly reduced while the changes significantly reduced mean annual costs of hospitalization. |
| White et al (2011) | UK | *n*280 | Number of PEG-related complications  Number of hospital admissions avoided  Number of replacement balloon gastrostomies | There were 343 PEG-related complications seen be the HEF team during this period.  103 patients required new balloon gastrostomies, of which 56 (43%) were performed as an emergency procedure.  228 hospital admissions were definitely avoided due to direct actions taken by the HEF team |  |
| White et al (2008) | UK | *n*180 | Number of PEG-related complications  Number of hospital admissions avoided  Number of replacement balloon gastrostomies | A total of 545 PEG-related complications were dealt  A total of 101 patients required new balloon retained gastrostomies), fifty-eight as emergency procedures (following PEG displacement, tube damage or blockage) and forty-three planned, with no complications. Although sixty-nine patients were admitted during this year, only fifteen (21%) were for PEG problems and all occurred at times of non-availability of staff at weekends or holidays or failure of carers to adhere to the written aftercare protocol. Hospital admissions were avoided in all fifty-eight instances (thirty patients, 12% of the total cohort of 245 patients) of PEG displacement, damage or blockage by emergency replacement by the HEF dietitians. | Hospital admissions were avoided in all fifty-eight instances of PEG displacement, damage or blockage by emergency replacement by the HEF dietitians.  However, it is likely that the early diagnosis and treatment of less urgent complications, and the training of patients, carers and other health care professionals will have avoided many further admissions. |
| Sanders et al (2001) | UK | *n*87  (*n*42 male) | Phone calls to endoscopy unit from  Number of home visits required  Nature of intervention required  Number of PEG-related admissions to hospital | During the 6-month study period, telephone advice was given 26 times with no further action required.  Home visits were necessary on 69 occasions.  There were 23 PEG-related emergency admissions accounting for 61 inpatient days however, all of these admissions were from nursing/residential homes, not patients living in their own home. | Prospective analysis of hospital support required and complications of PEG patients following discharge to the community.  Patients were given initial education after PEG insertion and then provided with a telephone number of the respective endoscopy unit for advice over the phone during normal working hours and a home visit if necessary. No routine follow-up service or dietitian review was provided after discharge. General practitioners were advised to refer patients with PEG problems to their respective endoscopy units.  During a 6-month period information was recorded by the Endoscopy Unit staff pertaining to the nature of these calls, the number of home visits required and any intervention that was necessary. In addition, any patients admitted with PEG-related complications were followed on a daily basis from the emergency admissions unit and their subsequent management noted. |

**SD:** standard deviation

**CI:** confidence interval

**1:** for total caseload of patients, *n* = 70

**2:** for 28 patients of caseload, based on number of admissions, bed days and day cases
